# Immunomodulatory Agents with Antivascular Activity in the Treatment of Non-Small Cell Lung Cancer: Focus on TLR9 Agonists, IMiDs and NGR-TNF

**DOI:** 10.1155/2010/732680

**Published:** 2010-06-03

**Authors:** Angelo Corti, Monica Giovannini, Carmen Belli, Eugenio Villa

**Affiliations:** ^1^Division of Molecular Oncology and IIT Network of Molecular Neuroscience, San Raffaele Scientific Institute University Hospital, 60 Olgettina St, 20132 Milan, Italy; ^2^Oncology Department, San Raffaele Scientific Institute University Hospital, 20132 Milan, Italy

## Abstract

Standard treatments for nonsmall cell lung cancer (NSCLC), such as surgery, chemotherapy, and radiotherapy, often lead to disappointing results. Unfortunately, also the various immunotherapeutic approaches so far tested have not produced satisfactory results to be widely applied in the clinical practice. However, the recent development of new immunomodulatory agents may open promising therapeutic options. This paper focuses on PF3512676, lenalidomide, and NGR-TNF, that is, drugs belonging to three different classes of immunomodulatory agents, that are also capable to affect tumor blood vessels with different mechanisms, and discusses the potential role of such agents in NSCLC treatment strategy.

## 1. Introduction

Lung cancer is the leading cause of cancer death in both men and women and one of the leading causes of death worldwide [1]. Non small cell lung cancer (NSCLC) represents ~80% of all types of lung cancer. Most patients present with locally advanced (stage III) or metastatic (stage IV) cancer [2, 3]. Despite chemotherapy treatment, sometimes in combination with radiotherapy, most patients die of disease progression, due to acquired or intrinsic resistance to chemotherapeutic drugs. Various immunotherapeutic approaches have been also attempted, ranging from the use of nonspecific immunostimulants like Bacillus Calmette-Guerin (BCG) to more specific strategies, unfortunately often with disappointing results [4, 5]. However, the recent improvement of our understanding of how the immune system works, the identification of new target antigens, and the development of new immunomodulators capable to affect the immune system and the tumor neovasculature could open new therapeutic options that deserve further investigation. 

This review discusses the potential role of three immunomodulatory agents that, besides regulating immune cells, can also affect the tumor neovasculature with different mechanisms and to different extent. In particular we discuss: (a) PF3512676, a Toll-like receptor 9 agonist primarily endowed of immunomodulatory activity, (b) lenalidomide, an immunomodulator with antiangiogenic properties and (c) NGR-TNF, an immunomodulatory agent with strong vascular damaging activity.

## 2. PF3512676 (TLR9 Agonist)

**Figure 1:**
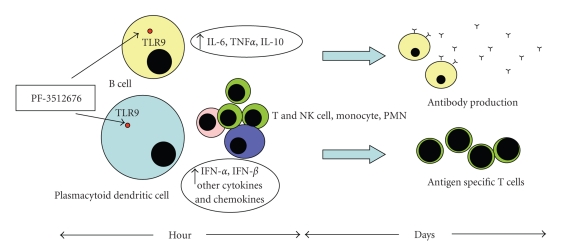
Proposed mechanism of action of PF-3512676. In the first phase, PF-3512676 activates a rapid induction of innate immune response through TLR9 stimulation in B cell and plasmocytoid cells. This may restore defective plasmocytoid dendritic cell function, induces IFN secretion (all IFN types), and activates NK and NKT cell. A later induction of adaptive immune response may then occur through dendritic cell mediated activation of antigen-specific T cells, monocytes and polymorphonuclear leukocytes, promotion of killer T cells and production of antibodies by B cells.

Toll like receptors (TLRs) are a family of highly conserved receptors that regulate innate antigen-specific immunity via the recognition of pathogen-associated molecular pattern [6–9]. TLR9 is expressed in endosomes of B and T lymphocytes, plasmocytoid cells, and dendritic cells [10]. Immunostimulatory oligonucleotides containing certain CpG sequence motifs stimulate the innate and adaptive immune response and have been under investigation for treating infectious diseases, allergies, asthma, and cancer [11–16]. Through TLR9 signalling pathway, immunostimulatory oligonucleotides activate a complex cascade that leads to increased production of proinflammatory cytokines and chemokines and stimulation of an immune response with antitumor effects [17–21]. Bioengineered immunomodulatory oligonucleotides have been developed to stimulate the immune system of various animal species, as well as purified and cultured human immune cells [22–24]. Several new immunomodulatory oligonucleotides have been evaluated in models of human cancer [25–27]. Among these, PF-3512676 (ProMune) is particularly promising. It contains unmethylated cytosine and guanine (CpG) motifs and a nuclease-resistant phosphorothioate backbone [28]. It is an agonist of the TLR9 expressed in plasmocytoid cells and B cells [28]. The anticancer activity of PF-3512676 is related to direct and indirect immunomodulation of both innate and adaptive immune responses [29]. Plasmocytoid dendritic cells stimulated by PF-3512676 express increased levels of MHC I and II and costimulatory molecules (leading to improved antigen presentation) secrete cytokines and chemokines that enhance natural killer (NK) cell activity directed toward tumor cells, present tumor specific antigens and costimulatory molecules to B and T cells, and generate long-living antigen specific cytotoxic T-lymphocytes and antibody responses [30]. A good indicator of activation and maturation of dendritic cells by PF-3512676 is the production of IFN*α* and the subsequent induction of interferon-inducible protein 10 (IP-10), an antiangiogenic cytokine [28, 31]. Furthermore upregulation of CD86 and CD80 on B cells induced by PF-3512676 and secretion of IL-10 and IL-6 demonstrate its strong stimulatory properties [30]. A schematic representation of the mechanism of action of PF-3512676 is shown in Figure 1. The clinical safety and efficacy of PF-3512676 have been evaluated in 18 clinical studies [30, 32]. Overall, 889 subjects were enrolled in these trials, where PF3512676 was administered via subcutaneous, intravenous, intralesional, or intramuscular routes as monotherapy or in combination with monoclonal antibodies or with chemotherapeutic agents. Immunological responses (such as induction of innate/adaptive immune responses with moderate to abundant cellular infiltrates of lymphocytes proven in tumor biopsies) leading in some cases to tumor regression were observed in patients with melanoma, renal cell carcinoma, non-Hodgkin lymphoma and non small cell lung cancer [28, 30, 33–38]. Focusing on NSCLC, a phase II study enrolling 112 chemonaive patients with NSCLC was conducted. The patients received PF3512676 in combination with platinum and taxane doublet chemotherapy at a dose of 0.2 mg/kg, sc, on the 2nd and 3rd weeks of a 3-week chemotherapy cycle [39, 40]. Twenty-eight (37%) patients had a partial or complete response with the combination of chemotherapy and PF-3512676 and 7 (19%) with chemotherapy alone. The combination of chemotherapy and PF-3512676 was well tolerated even if there was an excess of myelosuppression. The most commonly reported PF-3512676 related events were reversible local injection reactions, such as erythema, pain, induration, warmth and swelling, or systemic flu-like symptoms (fatigue, pyrexia, headache, chills, arthralgia and myalgia). Based on these preliminary data, two phase III trials were conducted to test the efficacy of PF-3512676 in combination with platinum based chemotherapy in advanced NSCLC patients. Unfortunately, both studies failed to prove the superiority of the combination after an interim analysis by an independent data monitoring safety committee [41], showing also a worse toxicity profile for the PF-3512676 arm [42, 43]. 

## 3. Lenalidomide (Immunomodulatory Imide Drug)

Another promising drug class among immunomodulatory agents is represented by Immunomodulatory imide Drugs (the so-called IMiDs). Thalidomide is the IMiDs progenitor. Second generation IMiDs are lenalidomide, pomalidomide and ENMD-0995. Thalidomide has emerged as a potent treatment for several disease entities. Although originally marketed in Europe as a sedative and antiemetic, reports of teratogenic effects [44] led to its withdrawal in the market in 1961 [45]. Thalidomide-associated congenital malformations were later explained as impaired vasculogenesis suggesting that a similar mechanism may contribute to prevent the growth of tumor blood vessels [46, 47]. In the cancer setting, thalidomide is currently used in multiple myeloma patients [48].

**Figure 2:**
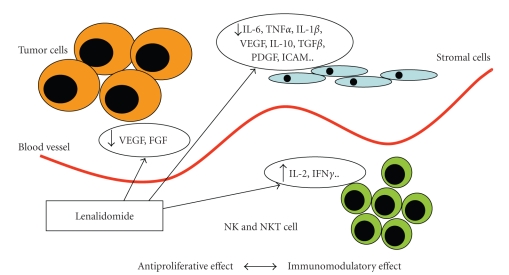
Proposed mechanism of action of lenalidomide. This drug can inhibit endothelial cell migration and adhesion possibly by downregulating endothelial cell integrins and angiogenesis. Lenalidomide can also induce immunomodulatory effect by activating T and NKT cells, which in turn release cytotoxic mediators, and by inhibiting the release of proinflammatory cytokines in the tumor microenvironment. The antiangiogenic effect is a major component of lenalidomide antitumor activity in solid tumors.

Lenalidomide is the first 2nd generation IMiD to be approved for clinical use. It has been registered as 2nd line treatment in association with dexamethasone in patients with multiple myeloma [49]. However, the therapeutic utility of this drug may not be limited to haematological malignancies. It was synthesized based on the structural backbone of thalidomide, by adding an amino group at position 4 of the phthaloyl ring and removal of the carbonyl group of the 4-amino-substituted phthaloyl ring. Such structural changes were designed to enhance its immunomodulatory and antitumor activity [49, 50]. Despite the proven clinical activity of the IMiDs the exact mechanism of their antitumor activity remains elusive. It is possible that the antitumor activity of lenalidomide is mediated through multiple nonmutually exclusive processes that primarily depend on the type of tumor cells and their microenvironment. Data on lenalidomide's mechanism of action, mostly derived from studies on multiple myeloma and B cell malignancies can likely be applied also to solid tumors. Lenalidomide has been shown to inhibit TNF-*α* [51, 52], IL-6 and other proapoptotic cytokines and proinflammatory mediators [49, 53], and to activate proapoptotic signals triggered by Fas-mediated cell death, such as caspase-8 (but not caspase-9) [49]. Lenalidomide downregulates antiapoptotic proteins like the cellular inhibitor of apoptosis protein 2 and FLICE inhibitor protein. Nuclear factor-kB is also directly inhibited by lenalidomide [49]. Lenalidomide is a potent stimulator of lipopolysaccharide-induced IL-10, as well as costimulators of T cells that are partially activated through the T-cell receptor, in the CD8+ subset [54, 55]. Furthermore, it induces increase in IL-2 and IFN*γ* secretion and upregulation of CD40L expression on anti-CD3 stimulated T cells, resulting in activation of natural killer cells, and thus improving host immunity against tumor cells [56]. Compared to thalidomide, lenalidomide is 50 to 2000 times more potent in stimulating T-cell proliferation and activation and 50–100 times more potent in augmenting IL-2 and IFN*γ* production [49]. In contrast with PF-3512676, which shows a predominant immunomodulatory activity, IMiDs are also proapoptotic agents and strong angiogenesis inhibitors. Indeed, various in vitro assays have demonstrated the antiangiogenic activity of IMiDs [57–60]. This activity is believed to be secondary to the inhibition of secretion of angiogenic cytokines, such as vascular endothelial growth factor (VEGF) and fibroblast growth factor (FGF), from both tumor and stromal cells. In addition, lenalidomide has been shown to inhibit endothelial cell migration and adhesion, perhaps by downregulating endothelial cell integrins [61, 62]. Lenalidomide is reported to downregulate key cytokines such as TNF-*α*, IL-6, IL-8 and VEGF, that is, cytokines which favour tumor cell survival, proliferation and, possibly, resistance to therapy, mainly by affecting the tumor vasculature [50]. A schematic representation of lenalidomide's mechanism of action is shown in Figure 2. In solid tumors, lenalidomide proved to have a good safety profile both in monotherapy and in combination with chemotherapy showing results in terms of antitumor activity in several tumor types and also in NSCLC [63–67]. In fact, Miller et al. tested the feasibility of lenalidomide at a dose escalated from 5 to 10 to 25 mg/day in 20 patients with solid tumors refractory to standard treatment [64]. Moderate dose-dependent and reversible haematological toxicity was observed. One partial response and three stable diseases were documented; of these patients three had NSCLC diagnosis. This study recommended 25 mg/day, orally, of lenalidomide as single agent for 4 weeks followed by 2-week rest period. Similarly, Kalmadi et al. explored safety and tolerability of lenalidomide in association with docetaxel and carboplatin in 14 patients with advanced solid tumors [67]. No treatment related deaths or irreversible toxicities were recorded. Five patients achieved partial response; 5 out of 9 patients had NSCLC. Docetaxel (60 mg/m^2^) and carboplatin (AUC 6) on day 1 with lenalidomide 5 mg daily orally for 2 weeks of a 21 day cycle was the maximum tolerated dose without the use of prophylactic growth factors. Clinically, lenalidomide shows a different and more manageable toxicity profile compared to thalidomide, causing greater haematological toxicity (neutropenia and thrombocytopenia) but much less neurological toxicity [49]. 

## 4. NGR-TNF

**Figure 3:**
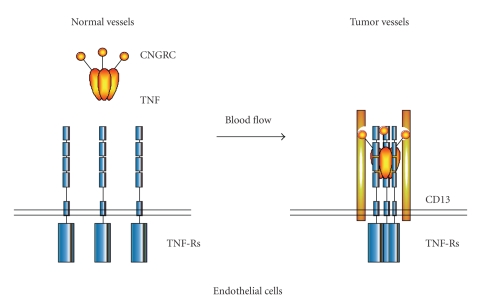
Proposed mechanism of the tumor homing properties of NGR-TNF. Ultra low dose NGR-TNF can interact more efficiently with CD13-positive tumor vessel, compared to CD13-negative normal vessels, by virtue of high avidity interactions of the CNGRC domain with CD13 and of the TNF domain with TNF receptors (TNF-Rs), thereby triggering local activation of endothelial cells, and inducing leukocyte adhesion molecules, cytokine secretion, procoagulant activity, and apoptosis.

NGR-TNF is an engineered TNF derivative with improved neovasculature homing properties. This drug, developed and initially tested at our Institute, is made of tumor necrosis factor *α* (TNF) fused to CNGRC, a peptide ligand of aminopeptidase N (CD13) overexpressed in tumor neovasculature [68–70]. A schematic representation of this drug and of the potential mechanism underlying its improved avidity for tumor blood vessels is shown in Figure 3. Experiments in animal models have shown that NGR-TNF, because of its tumor vasculature homing properties, is endowed of greater therapeutic activity and lower toxicity than TNF, enabling systemic administration of therapeutic doses [68, 71]. NGR-TNF can promote antitumor responses primarily by damaging the tumor vasculature. This provides the rationale for using NGR-TNF as a single agent. In addition, it has been demonstrated that ultra-low doses of NGR-TNF (picograms/mouse) are sufficient to alter permeability in tumor vessels of tumor-bearing mice and improve the penetration of various chemotherapeutic drugs in tumor tissues, including melphalan, doxorubicin, cisplatin, gemcitabine, and paclitaxel [71–73]. Thus, NGR-TNF may have a dual pharmacological effects, acting both as a vascular damaging agent and as an enhancer of chemotherapy. In principle, NGR-TNF could be exploited either to improve the penetration of conventional doses of chemotherapeutic drugs in tumors, favoring their local antitumor and immune adjuvant effects, or to reduce the dose of chemotherapeutic drugs and their toxicity, including that against cells of the immune system. This combined strategy might also benefit from the ability of TNF to promote anti-tumor immune responses [74, 75]. Interestingly, targeted delivery of TNF alone or in association with chemotherapy has been shown to cure tumors in animal models and to induce protective immunity [68]. This suggests that the immune response, and in particular T-cell dependent mechanisms, represent an important arm of NGR-TNF activity. TNF targeted to vessels might also enhance the production of endothelial immuneregulating cytokines or chemokines and/or upregulate endothelial adhesion molecules, favoring extravasation of immune cells, and improving the ability of the immune system to cope with residual tumor cells [76]. A schematic representation of NGR-TNF mechanism of action is shown in Figure 3.

Various Phase I and Phase II clinical studies have been undertaken with NGR-TNF in solid tumors showing manageable toxicity profile and evidence of disease control, particularly in hepatocarcinoma, pleural mesothelioma, and colorectal cancer [77–80]. At our institution, we are currently testing low-dose NGR-TNF both as a single agent and in combination with chemotherapeutic agents, such as antracyclines and cisplatin, in several solid tumors, such as pleural mesothelioma, NSCLC and small cell lung cancer. Further studies are warranted to confirm NGR-TNF as a treatment option for NSCLC patients.

## 5. Conclusions

Standard treatment options for NSCLC patients, who are mostly diagnosed in advanced stage of disease, often lead to disappointing results. Immunotherapy is a promising approach in several tumors and preliminary but promising data are arising also for lung cancer. Despite initial attempts to treat NSCLC with immunomodulatory agents were unsuccessful, the development of new drugs endowed of immunomodulatory and antivascular activity have stimulated further clinical studies. The first agent we focused on (PF-3512676) is an example of an immunomodulatory agent with modest antivascular activity. Unfortunately, also this drug eventually turned out to be ineffective in large phase III trials for advanced NSCLC. However, the promising early findings, in terms of efficacy and toxicity, obtained with lenalidomide and NGR-TNF, that is, two immunomodulatory agents endowed with strong antivascular activity, may suggest that these compounds could play a role in the treatment of NSCLC, both as single agents and in combination with chemotherapeutic drugs. These combinations could be the key to move a step forward in improving prognosis in NSCLC patients.
